# Supplementary material for: WordCommentsAnalyzer: A windows software tool for qualitative research
Source: F1000Res. 2018 Sep 4;7:536. Originally published 2018 May 3. [Version 2] doi: 10.12688/f1000research.14819.2 (PMC6137411; doi:10.12688/f1000research.14819.2)
Supplement: Supplementary file 3 [file f1000research-7-17614-s0002.tgz › b2a79496-4ada-47c4-9682-a99ce867836d.zip!/Eight Iranian Health Journals/Journal of Research in Rehabilitation Sciences _37.docx]

© Journal of Research in Rehabilitation Sciences . This work is licensed under CC BY-NC 4.0 (https://creativecommons.org/licenses/by-nc/4.0/).

Volume & Issue: دوره 8، شماره 4: 1391

Title: تأثير كفي با گوه خارجي پاشنه بر استئوآرتريت كمپارتمنت

http://jrrs.mui.ac.ir/index.php/jrrs/article/view/351

مقدمه :استئوآرتريت زانو يكي از علل شايع زانو درد در سالمندان بوده كه باعث درد و اشكال راه رفتن در آن‌ها مي‌شود و شيوع آن در زنان بيشتر از مردان می‌باشد. شيوع اين عارضه در كمپارتمنت داخلي بيشتر است و اغلب منجر به تخريب فضاي داخلي مفصل مي‌شود. با تجويز كفي با گوه خارجي خط وزن از روي بخش داخلي به سمت خارج منتقل شده و در نتيجه باعث كاهش فشار روي سمت داخل زانو و كاهش درد مي‌شود .هدف از این مطالعه، بررسي ميزان تأثير كفي با گوه خارجي پاشنه در افراد مبتلا به استئوآرتريت كمپارتمنت داخلي زانو بود. مواد و روش‌ها: در اين مطالعه 36 بيمار مبتلا به استئوآرتريت كمپارتمنت داخلي زانو پس از گذراندن مراحل غربالگري به دو گروه 18 نفره تقسيم شدند. گروه اول از كفي با گوه خارجي 3 ميلي‌متر در پاشنه و گروه دوم از كفي با گوه خارجي 7 ميلي‌متر در پاشنه استفاده كردند. ميزان درد و كيفيت زندگي بيماران با استفاده از پرسش‌نامه‌هاي تنظيم شده و بر اساس سيستم WOMAC و شدت استئوآرتريت و ميزان زاويه تيبيوفمورال با استفاده از عكس راديوگرافي اندازه‌گيري شد . يافته‌ها: ميزان درد، كيفيت زندگي و زاويه تيبيوفمورال به طور معني‌داري بهبود يافت؛ در حالي كه شدت استئوآرتريت پس از استفاده از كفي تفاوتي با قبل نداشت. در بين دو گروه كفي‌هاي 7 ميلي‌متر به طور معني‌داري نسبت به كفي 3 ميلي‌متر باعث كاهش درد شدند . نتيجه‌گيري :طبق نتايج به دست آمده از اين تحقيق، استفاده از كفي با گوه خارجي پاشنه باعث تصحيح زاويه تيبيوفمورال، كاهش درد و افزايش كارايي مفصل زانو مي‌شود .

Title: بررسی پایایی نسخه فارسی ابزار سنجش شدت لکنت- ویرایش چهارم ویژه بزرگسالان مبتلا به لکنت

http://jrrs.mui.ac.ir/index.php/jrrs/article/view/285

مقدمه: سنجش شدت لکنت یک جنبه مهم و اساسی در انجام هر گونه اقدام پیرامون لکنت است. یکی از ابزارهای مهم و معتبر در سنجش شدت لکنت 4SSI می‌باشد. این ابزار امروزه به عنوان سنجش بخشی از لکنت در مقالات و همچنین ارزیابی نتایج درمان استفاده می‌شود. با توجه به این که ارزیابی نتایج درمان، یکی از بخش‌های مهم درمان مبتنی بر شواهد است و با در نظر گرفتن این موضوع که وجود و کاربرد این ابزارها در کشور ما بسیار محدود می‌باشند، بر آن شدیم که در این مطالعه به بررسی پایایی این ابزار معتبر و شناخته شده در زمینه سنجش لکنت بپردازیم. مواد و روش‌ها: این مطالعه از نوع توصیفی- تحلیلی و مقطعی می‌باشد. بعد از ترجمه چند مرحله‌ای آزمون مطابق با پروتکل IQOLA و بررسی روایی آن، پایایی ابزار بر روی 35 فرد بزرگسال مبتلا به لکنت با استفاده از 5 آزمونگر باتجربه مورد بررسی قرار گرفت. یافته‌ها: پایایی‌ها در این مطالعه با استفاده از روش‌های آماریICC ، One-way repeated measures ANOVA و Cronbach¢s alpha مورد بررسی قرار گرفت. پایایی آزمون در حالت میان آزمونگر با استفاده از روش‌های  Cronbach¢s alphaو ICC برای هر پارامتر، 98/0- 93/0 و پایایی درون آزمونگر با استفاده از روش‌های آماری ICC و one- way repeated measures ANOVA برای هر پارامتر، 98/0- 85/0 گزارش گردید. نتیجه‌گیری: با توجه به پایایی بسیار بالای به دست آمده در این مطالعه، می‌توان نتیجه گرفت که نسخه فارسی آزمون 4SSI برای ارزیابی شدت لکنت در نمونه‌های فارسی زبان مناسب و بسیار کارامد است.

Title: تأثير يک دوره برنامه تمريني منتخب بر کيفيت زندگي زنان سالمند مبتلا به پوکي استخوان

http://jrrs.mui.ac.ir/index.php/jrrs/article/view/368

مقدمه: پوكي استخوان يک بيماري رايج و يكي از مشكلات اساسي سيستم استخوان‌بندي افراد سالمند محسوب مي‌شود. در افراد مسن، کاهش در تراکم مواد معدني استخوان (پوکي استخوان) اغلب با کاهش کيفيت زندگي همراه است. بنابراين هدف اصلي اين پژوهش، ارزيابي تأثير يک دوره برنامه تمريني منتخب بر کيفيت زندگي زنان سالمند مبتلا به پوکي استخوان بود. مواد و روش‌ها: تعداد 27 زن مبتلا به پوکي استخوان (با میانگین سنی 6 ± 58 سال) مراجعه کننده به مرکز سنجش پوکي استخوان اصفهان به عنوان نمونه تحقيق انتخاب و در دو گروه تجربي (14 نفر) و شاهد (13 نفر) قرار گرفتند. در ابتدا کيفيت زندگي بيماران توسط پرسش‌نامه کيفيت زندگي (41- QUALEFFO) مورد ارزيابي قرار گرفت و سپس گروه تجربي در برنامه تمريني منتخب شامل تمرينات دامنه حرکتي، مقاومتي و تعادلي به مدت 8 هفته، 3 بار در هفته شرکت کرد. گروه شاهد در اين مدت تحت هيچ مداخله‌اي قرار نگرفت. بعد از برنامه تمريني دوباره کيفيت زندگي افراد مورد ارزيابي قرار گرفت. جهت تجزيه و تحليل داده‌ها، از نرم‌افزار SPSS و آزمون‌هاي Independent t و Paired t استفاده شد. يافته‌ها: تفاوت معنی‌داري (05/0 > P) در ميانگين درد (17 ± 7/30 در مقابل 11 ± 4/15)، تحرک (8 ± 1/23 در مقابل 7 ± 6/17) ، فعاليت اجتماعي (9/12 ± 9/27 در مقابل 8 ± 2/20)، درک سلامت عمومي (21 ± 2/51 در مقابل 16 ± 35)، عملکرد ذهني (6 ± 8/20 در مقابل 5 ± 1/15) قبل و بعد از برنامه تمريني در گروه تجربي مشاهده شد، اما در بخش‌هاي مربوط به کار خانه و فعاليت روزانه  تفاوت معنی‌داري (05/0 > P) مشاهده نشد. در گروه شاهد در هيچ يک از متغيرها تفاوت معنی‌داري مشاهده نشد (05/0 > P). بين گروه تجربي و شاهد در كليه متغيرها در پيش آزمون تفاوت معنی‌داري مشاهده نشد (05/0 < P)، اما در پس‌آزمون تفاوت معنی‌داري مشاهده شد (05/0 < P).               نتيجه‌گيري: به نظر مي‌رسد تمرينات دامنه حرکتي، مقاومتي و تعادلي در بهبود کيفيت زندگي بيماران مبتلا به پوکي استخوان تأثير دارد. بنابراين توصيه مي‌شود اين گونه تمرينات در برنامه زندگي افراد مبتلا به پوکي استخوان در نظر گرفته شود.

Title: رویکرد جانشینی الکتریکی- لامسه‌ای به عنوان راهكاري نوين در توان‌بخشی دهليزي- مطالعه اولیه

http://jrrs.mui.ac.ir/index.php/jrrs/article/view/327

مقدمه: تاكنون بيماران مبتلا به افت دهليزي دو طرفه ناشی از اتوتوكسيستي با آمينوگلوكوزيدها، با روش‌هاي مرسوم توان‌بخشي شامل تمرينات تطابقي و عادت‌سازي مورد توان‌بخشي قرار مي‌گرفتند. مطالعات نشان داده كه روش توانبخشی دهلیزی توسط سیستم جانشینی الکتریکی-لامسه‌ای (Electrotactile vestibular  substitution system یا EVSS) مي‌تواند نقش مهمي در روند توان‌بخشي اين بيماران داشته باشد. مطالعه حاضر یک مطالعه اولیه به منظور بررسي تأثير اين مداليته درماني می‌باشد.مواد و روش‌ها: در این پژوهش، 6 مرد و 4 زن مبتلا به افت دهلیزی دو طرفه، مورد توان‌بخشی توسط سيستم EVSS قرار گرفتند. این توان‌بخشی طی 10 جلسه درمانی 20 دقیقه‌ای (روزی 2 جلسه) انجام شد. پروتکل آزمون سازماندهی حسی و پرسشنامه سرگیجه جهت مقایسه نتایج قبل و بعد از درمان مورد استفاده واقع شدند. آزمون‌های پس از درمان، یک روز پس از اتمام درمان EVSS انجام گرفتند. میزان اختلاف بین گروه‌های قبل و بعد از درمان، توسط آزمون t با استفاده از نرم افزار آماری 16SPSS با لحاظ قرار دادن شرط اختلاف معنی‌داری (05/0 > P) بررسی شد.یافته‌ها: همه بیماران پس از دوره 5 روزه درمان توسط EVSS در SOT و پرسش‌نامه DHI امتیازات بهتری کسب کردند (05/0 > P). دفعات افتادن در مرحله 5 SOTکاهش یافت (05/0 > P). بیماران پس از این دوره قادر بودند وضعیت عمودی خود را در موقعیت رومبرگ با چشمان بسته و روی سطح نرم حفظ کنند و حرکات یکنواخت تر و راه رفتن (Gait) پایدارتر شد (05/0 > P).نتیجه‌گیری: این نتایج نشان دهنده بهبود علایم بیماران به واسطه رویکرد جانشینی الکتریکی-لامسه‌ای می‌باشد که بر امکان جانشینی سیستم‌های حسی جهت بهبود نقایص دهليزي تأکید دارد.

Title: نقش منبع کنترل و ابعاد شخصیت در پیش‌بینی رضایت شغلی کاردرمانگران مراکز دولتی شهر تهران

http://jrrs.mui.ac.ir/index.php/jrrs/article/view/497

مقدمه: هنگام بررسی رضایت شغلی، توجه به روحیه و انگیزه‌های انسانی افراد شاغل از اولویت بالایی برخوردار است. این پژوهش با هدف، بررسی نقش منبع کنترل و ابعاد شخصیت در پیش‌بینی رضایت شغلی کاردرمانگران مراکز دولتی شهر تهران انجام شد. مواد و روش‌ها: در پژوهش حاضر 75 نفر کاردرمانگر (23 مرد و 52 زن) که سن آن‌ها بین 23 تا 50 سال (00/7 ± 37/32 =M) بود و در مراکز دولتی اشتغال داشتند، شرکت کردند. برای جمع‌آوری داده‌های پژوهش از پرسش‌نامه‌های فرم کوتاه، پرسش‌نامه شخصیتی نئو، منبع كنترل دروني بيروني راتر و پرسش‌نامه رضايت شغلي هرزبرگ استفاده شد. داده‌های پژوهش از طریق آزمون‌های آماری همبستگی Pearson و آزمون رگرسیون و با استفاده از نرم‌افزار 18SPSS تحلیل شد. یافته‌ها: یافته‌های تحلیل همبستگی Pearson نشان داد که بین جایگاه کنترل (001/0 = P) و چهار بعد شخصیت یعنی ابعاد روان‌نژندی (001/0 > P)، برون‌گرایی (009/0 = P)، توافق (012/0 = P) و وجدانی بودن (024/0 > P) با رضایت شغلی کاردرمانگران همبستگی معنی‌داری وجود دارد. بین گشودگی (Openness) (061/0 = P) و رضایت شغلی کاردرمانگران همبستگی معنی‌داری مشاهده نشد. نتایج تحلیل رگرسیون نشان داد که تنها ضریب بتای بعد شخصیت روان‌نژندی (009/0 = P) و جایگاه کنترل (047/0 > P) در پیش‌بینی رضایت شغلی کاردرمانگران نقش معنی‌دار دارد. نتیجه‌گیری: برای افزایش رضایت شغلی کاردرمانگران توجه به جایگاه کنترل و بعد شخصیت روان‌نژندی آن‌ها بیشترین اهمیت را دارد.

Title: تأثير ارتوزهاي پيش ساخته مچ پا بر تعادل ورزشكاران مبتلا به بي‌ثباتي مزمن مچ پا در زمان خستگی

http://jrrs.mui.ac.ir/index.php/jrrs/article/view/726

مقدمه: حدود 28-10 درصد آسیب دیدگی‌های ورزشی را پیچ خوردگی مچ پا تشکیل می‌دهد. بيشتر افرادي كه دچار اسپرين خارجي مچ پا مي‌شوند، به طور كامل بهبود نمي‌يابند و به طور تقريبی 80 درصد از بازيكنان بسكتبال از در رفتگي‌هاي مكرر مچ پا رنج مي‌برند. اختلال در تعادل، يكي از رایج‌ترین مشكلاتي است كه براي اين افراد آسيب ديده بروز مي‌كند. علاوه بر آن، پس از مدتي رقابت ورزشي، ورزشكار دچار خستگي می‌شود و در نتیجه مشكلاتي كه به دنبال خستگي به وجود مي‌آيند و همچنین مشكلات ناشي از بي‌ثباتي مزمن مچ پا، خطر بروز اسپرين مجدد را افزايش مي‌دهد. با توجه به آن که استفاده از ارتوزها یکی از ابزارهای جلوگیری از آسیب است، هدف تحقیق حاضر، بررسی تأثیر ارتوزها بر تعادل ورزشكاران مبتلا به بي‌ثباتي مزمن مچ پا در زمان خستگي بود مواد و روش‌ها: 20 بسکتبالیست مبتلا به بی‌ثباتی مزمن مچ پا و 15 بسکتبالیست سالم ابتدا در شرایط عادی و سپس در زمان خستگی، مورد ارزیابی تعادل با استفاده از دستگاه بایودکس، در سه وضعيت بدون استفاده از ارتوز، با انكل ساپورت ساده و نیز با انکل ساپورت فنري قرار گرفتند. برای بررسي تأثير ارتوزهاي پري‌فابريك از آزمون Repeated measures ANOVA استفاده شد و سطح معني‌داري 05/0 > P در نظر گرفته شد. یافته‌ها: نتايج حاصل از تجزيه و تحليل آماري داده‌ها نشان داد كه ارتوزهای پری‌فابریک بر تعادل ورزشكاران سالم و مبتلا به بي‌ثباتي مزمن مچ پا در زمان خستگي تأثيرگذار بوده‌اند. ‎نتیجه گیری: با توجه به تأثير مثبت ارتوزها بر تعادل ورزشکاران در زمان خستگی، می‌توان از ارتوزها برای جلوگيري از بروز آسيب ديدگي در حین تمرین و در مسابقات ورزشی استفاده نمود.

Title: اثربخشی تقویت حافظه دیداری به طریق بازی درمانی بر کاهش خطاهای املایی دانش‌آموزان دارای اختلال نوشتن

http://jrrs.mui.ac.ir/index.php/jrrs/article/view/585

مقدمه: در ارتباط با نقایص شناختی دانش‌آموزان مبتلا به اختلال نوشتن فرضیات متعددی وجود دارد که یکی از این فرضیات نقص در حافظه دیداری است. هدف از مطالعه حاضر، اثربخشی تقویت حافظه دیداری به طریق بازی درمانی بر کاهش خطاهای املایی دانش‌آموزان دارای اختلال نوشتن بود. مواد و روش‌ها: این پژوهش از نوع شبه آزمایشی ( پیش‌آزمون- پس‌آزمون با گروه شاهد) بود. در این مطالعه، 30 دانش‌آموز مبتلا به اختلال نوشتن از بین کل دانش‌آموزان دختر و پسر مراجعه کننده به مرکز اختلالات یادگیری شهرستان دیواندره به کمک ابزارهای تشخیصی، آزمون Wechsler، آزمون نوشتن فلاح چای و ارزیابی غلط‌های املایی انتخاب شدند، سپس به طور تصادفی در دو گروه آزمایش (15 نفر) و شاهد ( 15 نفر) قرار گرفتند و سپس گروه آزمایش به مدت 20 جلسه و طی حدود 3 ماه تحت آموزش و تقویت حافظه دیداری به طریق بازی درمانی قرار گرفت و در طول جلسات درمانی گروه آزمایش، گروه شاهد هیچ مداخله‌ای دریافت نکرد. داده‌های جمع‌آوری شده با استفاده از آزمون ANOVA، ANCOVA تک متغیری، آزمون همبستگی Pearson و Independent t تحلیل شد. همچنین داده‌ها با نرم‌افزار 16SPSS پردازش گردید. یافته‌ها: F تک متغیری (حافظه دیداری = 05/0 > P، آزمون مکعب‌ها =05/0 > P) نمره‌های پس‌آزمون گروه آزمایش و شاهد نشان داد که دو گروه در حافظه دیداری و خرده آزمون مکعب Wechsler از لحاظ آماری با یکدیگر تفاوت معنی‌داری دارند. همچنین نتایج آزمون Independent t نشان داد که میانگین  نمرات حافظه دیداری و آزمون مکعب‌ها در مرحله پس‌آزمون به لحاظ جنسیت تفاوت معنی‌داری با یکدیگر ندارند. نتیجه‌گیری: تقویت حافظه دیداری دانش‌آموزان دارای اختلال نوشتن باعث کاهش خطاهای املایی آن‌ها خواهد شد. این مداخله باعث شد دانش‌آموزان بر فرايند نوشتن خود نظارت كنند و فعالیت خود را هدفمند سازند و در نهایت مهارت نوشتن صحیح کلمات را کسب کنند.

Title: بررسی مقایسه‌ای مهارت‌های نحوی کودکان کم‌شنوا و شنوای فارسی زبان پیش دبستانی

http://jrrs.mui.ac.ir/index.php/jrrs/article/view/436

مقدمه: بررسی مهارت‌هاي نحوي کودکان آسيب ديده شنوايي از حوزه‌هاي مورد توجه آسيب‌شناسان گفتار و زبان است. در اين مطالعه     ميانگين طول گفته، نوع و تعداد الگوهاي خطاي گفتار كودكان كم‌شنوا در دو نوع نمونه گفتار ( آزاد و توصيفي) با همتايان شنوايشان مقايسه شد. مواد و روش‌ها: در این پژوهش مقطعی- توصیفی- تحلیلی، 16 کودک شنوا و 9 کودک کم‌شنوای 48 تا 72 ماهه تک زبانه و فارسی زبان از دو مرکز توان‌بخشی و مهدهای کودک شهر تهران به روش نمونه‌گیری در دسترس انتخاب شدند. داده‌های گفتار توصیفی با روش بازگویی داستان و توصیف تصویر و داده‌های گفتار آزاد از طریق پرسش‌های مکمل جمع‌آوری شد. تحلیل زبانی داده‌های جمع‌آوری شده، به دو روش كمي و كيفي مبتني بر صداي ضبط شده صورت پذيرفت. تحلیل آماری داده‌هاي به دست آمده با استفاده از آزمون‌هاي پارامتريك و غیر پارامتريك و Mann-Whitney U و t انجام شد. يافته‌ها: تحلیل داده‌هاي زباني بین دو گروه کم‌شنوا و شنوا حاكي از تفاوت معنی‌دار میانگین طول گفته (MLU) بر حسب تکواژ در هر دو نوع گفتار آزاد و توصیفی بوده (به ترتیب 002/0 = P و 001/0 > P). مقایسه میانگین كل خطاها هم در گفتار آزاد و هم در گفتار توصيفي بین دو گروه تفاوت معنی‌داری نشان داد (به ترتیب 003/0 = P و 001/0 > P). در گفتار توصیفی کودکان کم‌شنوا بیشترین خطای دستوری حذف نادرست و در کودکان شنوا انتخاب نابجا بود (به ترتیب 5/48 درصد و 2/38 درصد) و در گفتار آزاد بیشترین خطای هر دو گروه شنوا و کم‌شنوا حذف نابجای دستوری بود (به ترتیب 4/47 درصد و 8/38 درصد). نتیجه‌گیری: در مطالعه بين گروهي کودکان کم‌شنوا علاوه بر MLU کوتاه‌تر به وي‍‍ژه در گفتار آزاد نسبت به شنواها از نظر داشتن نوع خطاهای صرفی و نحوی نیز با آنان تفاوت معنی‌داری نشان دادند.

Title: مقايسه يادگيري يك الگوي حركتي دست در بزرگسالان جوان 20 تا 26 ساله و كودكان 8 تا 10 ساله و كاربرد آن در توان‌بخشي

http://jrrs.mui.ac.ir/index.php/jrrs/article/view/447

مقدمه: هدف از مطالعه حاضر، مقايسه يادگيري يك الگوي حركتي دست كه مستلزم هماهنگي فضايي و زماني بود، در دو گروه سني كودكان و بزرگسالان جوان بود. مواد و روش‌ها: 60 آزمودني بنا بر سن (كودك- بزرگسال) و شيوه ارایه بازخورد (100 درصدي، حذفي، خودكنترل) به طور تصادفي در 6 گروه تمريني قرار گرفتند. در روزهاي اول و دوم، در هر روز آزمودني‌ها به تعداد 100 كوشش به تمرين تكليف مورد نظر پرداختند و در طي روزهاي دوم و سوم آزمون‌هاي يادداري و در روز سوم آزمون بازاكتساب به عمل آمد. جهت پردازش داده‌ها از آزمون‌هايANOVA  با اندازه‌گيري‌هاي مكرر و ANOVA يك سويه استفاده شد (05/0 ≥ P). یافته‌ها: در كوشش‌هاي تمريني روز اول بر دقت تمامي آزمودني‌ها به شكل معنی‌داري افزوده شد، اما در روز دوم تنها بر دقت عملكرد بزرگسال جوان افزوده شد. بين آزمون‌هاي يادداري كودكان تفاوت معنی‌داري مشاهده نشد؛ در حالي كه اين امر در مورد بزرگسالان جوان معنی‌دار بود. گروه بازخوردي 100 درصدي كودكان در آزمون‌هاي يادداري عملكرد بهتري از ديگر گروه‌هاي بازخوردي كودكان داشت، اما در گروه‌هاي بازخوردي بزرگسالان جوان تفاوت معنی‌داري مشاهده نشد. گروه بازخوردي 100 درصدي كودكان در آزمون بازاكتساب عملكرد بهتري از ديگر گروه‌هاي بازخوردي كودكان داشت، اما در گروه‌هاي بازخوردي بزرگسالان جوان تفاوت معنی‌داري مشاهده نشد. نتیجه‌گیری: نتايج حاصل از اين مطالعه نشان داد، بزرگسالان جوان در فراگيري اين الگوي حركتي بهتر از كودكان عمل مي‌كنند. ارایه بازخورد بيشتر به كودكان مي‌تواند سبب ارتقای يادگيري حركتي كودكان شود (مغاير با فرضيه هدايت) و نبايد انتظار داشت كودكان در كسب مهارت‌هاي حركتي همانند بزرگسالان پيشرفت نمايند.

Title: اثربخشی برنامه توان‌بخشی روانی- حرکتی بر بهبود مهارت‌های روانی- حرکتی دانش‌آموزان دبستانی عقب مانده ذهنی آموزش‌پذیر

http://jrrs.mui.ac.ir/index.php/jrrs/article/view/577

مقدمه: مهارت‌های روانی- حرکتی نقش مهمی در یادگیری تحصیلی و جنبه‌های رشد کودکان دارد. بر همین مبنا هدف از پژوهش حاضر، تعیین میزان اثربخشی برنامه توان‌بخشی روانی- حرکتی بر بهبود مهارت‌های روانی- حرکتی دانش‌آموزان دبستانی عقب مانده ذهنی آموزش‌پذیر بود. مواد و روش‌ها: جامعه آماری این تحقیق را دانش‌آموزان دبستانی دختر عقب مانده ذهنی آموزش‌پذیر شهر اصفهان تشکیل دادند که از میان آنان 30 نفر به روش نمونه‌گیری تصادفی خوشه‌ای چند مرحله‌ای انتخاب شدند. روش پژوهش، آزمایشی از نوع پیش آزمون- پس‌آزمون با گروه شاهد بود. ابزار پژوهش مقیاس رشد حرکتی Lincoln- avartsy بود. داده‌ها در سطح توصیفی با میانگین و انحراف استاندارد و در سطح استنباطی با ANCOVA مورد تجزیه و تحلیل قرارگرفت. یافته‌ها: برنامه توان‌بخشی روانی- حرکتی بر بهبود مهارت‌های روانی- حرکتی مؤثر بود (001/0 > P). همچنین این برنامه بر برخی از مؤلفه‌های روانی- حرکتی مانند مهارت انگشتان، راه رفتن عقب عقب نیز تأثیر داشته است (05/0 > P). نتیجه‌گیری: با توجه به نتایج به دست آمده از این پژوهش، می‌توان نتیجه گرفت که انجام فعالیت‌های روانی- حرکتی به عنوان بخش ضروری و اصلی برنامه روزانه این کودکان می‌تواند نتایج پرثمرتری برای آن‌ها به همراه داشته باشد.

Title: تأثير دو روش تمريني ريباندتراپي و تمرينات هوازي بر روي ظرفيت هوازي، سطح اندوتلين پلاسما و کيفيت زندگي مردان مبتلا به آسم

http://jrrs.mui.ac.ir/index.php/jrrs/article/view/549

مقدمه: آسم از شايع‌ترين اختلالات دستگاه تنفسي است. تمرينات ورزشي يکي از اجزای اساسي و مهم بازتواني بيماران ريوي مي‌باشد. اين تحقيق با هدف، بررسي و مقايسه تأثير دو روش تمريني ريباندتراپي و تمرينات هوازي روي بيماران مبتلا به آسم صورت گرفت. مواد و روش‌ها: تعداد 26 نفر بيمار از ميان بيماران مبتلا به آسم، مراجعه کننده به مرکز درماني ريوي بيمارستان الزهراي اصفهان انتخاب و به صورت تصادفي در دو گروه ریباندتراپی و هوازی قرار گرفتند. تمامي بيماران مرد بوده و در محدوده سني 20 تا 40 سال قرار داشتند. ظرفيت هوازي (VO2max) با آزمون Bruce تعدیل شده تعیین شد. سطح اندوتلين-1 سرم به روش ELISA و کيفيت زندگي با پرسش‌نامه استاندارد كيفيت زندگي بيماران مبتلا به آسم (AQLQ) اندازه‌گيري گرديد. پس از اخذ رضايت‌نامه، بيماران به مدت 8 هفته و در هر هفته دو جلسه به تمرينات منتخب تحت نظر متخصص پرداختند. جهت تحليل داده‌ها از روش آماري ANOVA براي داده‌هاي تکراري در سطح معنی‌داری 05/0 > P استفاده شد. يافته‌ها: تغييرات درون گروهي (پس‌آزمون نسبت به پیش‌آزمون) و همچنين روند این تغييرات (شيب خط تغييرات) در تمامي متغيرها به نفع گروه ریباندتراپی معنی‌دار بود (05/0 > P). تفاوت دو گروه در متغيرهاي اندوتلين و ظرفيت هوازي تحقيق معنی‌دار نبود (به ترتیب 80/0 = P و 06/0 = P)، ولي در متغير کيفيت زندگي معنی‌دار بود (01/0 = P). نتيجه‌گيري: به نظر مي‌رسد تمرينات ريباندتراپي بتواند به عنوان روش مکمل مفيدي در کاهش برخي مشکلات بيماران مبتلا به آسم معرفي و مورد استفاده قرار گيرد.

Title: توصیف برخي ساختارهاي زباني در داستان‌گويي كودكان 4 ساله سالم شهر اصفهان و ارتباط آن با جنس بر اساس پروتكل ارزيابي داستان‌گويي

http://jrrs.mui.ac.ir/index.php/jrrs/article/view/427

مقدمه: مهارت داستان‌گویی کودکان به عنوان يك پايه مهم و پيش‌بيني كننده اكتساب مهارت‌هاي خواندن، نوشتن، تحصيلي و اجتماعی است. ارزیابی داستان رویکردی غنی برای توصیف رشد زبان در محیط‌های اولیه کودکی می‌باشد. این پژوهش با هدف، توصیف برخی از ساختارهای زبانی در داستان‌گویی کودکان 4 ساله سالم و ارتباط آن با جنس بر اساس پروتکل ارزیابی داستان‌گویی انجام شد. مواد و روش‌ها: در این مطالعه توصيفي- تحليلي و مقطعی، برخی از ساختارهای زبانی 126 كودك فارسي زبان 4 ساله سالم شهر اصفهان، در داستان‌گویی خودانگیخته با کمک یک کتاب مصور بر اساس شیوه پروتکل ارزیابی داستان‌گویی مورد بررسی قرار گرفتند. پایایی‌های درون آزمونگر، بین آزمونگر و پايايي بين نمره‌گذاري همزمان از روي نمونه صوتي ضبط شده و نمونه نسخه‌نویسی شده، نیز انجام شد. یافته‌ها: ساختارهای زبانی مورد نظر در داستان‌های اغلب کودکان وجود داشت. بیشترین رخداد مربوط به صورت ملکی اسم و کمترین استفاده از کاربرد مجازی اسم بود. نمرات پایایی‌های درون آزمونگر، بین آزمونگر و پايايي بين نمره‌گذاري همزمان از روي نمونه صوتي ضبط شده و نمونه نسخه‌نویسی شده بین 53/0 تا 00/1 بود. در نمره نهایی و نمرات هر یک از ساختارهای زبانی، تفاوت معنی‌داری بین دختران و پسران وجود نداشت. نتیجه‌گیری: داده‌های مطالعات گذشته در مورد دامنه میانگین طول گفته در سنین مختلف و نتایج ذکر شده از ارتباط بین میانگین طول گفته و رشد ساختارهای نحوی زبان در محدوده سنی مورد نظر، با یافته‌های تحقیق حاضر تطابق داشت.

Title: تأثیر روند ایجاد خستگی بر روی ثبات وضعیتی و در حین انجام حرکات تکراری تنه در دو گروه افراد سالم و بیماران کمردرد غیر اختصاصی

http://jrrs.mui.ac.ir/index.php/jrrs/article/view/456

مقدمه: مطالعه حاضر یک مطالعه مورد شاهدی از نوع شبه تجربی بود که به بررسی تأثیر خستگی بر روی کنترل تعادل و ثبات وضعیتی در افراد کمردردی و گروه سالم پرداخت. مواد و روش‌ها: 14 فرد سالم و 13 بیمار کمردردی حرکات تکراری خم و راست شدن تنه را در سطح میدساژیتال تا سر حد خستگی انجام دادند. پارامترهای استخراجی از مرکز فشار که به عنوان شاخصی جهت ارزیابی تعادل و ثبات وضعیتی هستند و در این مطالعه مورد استفاده قرار گرفتند شامل انحراف معیار دامنه حرکت، انحراف معیار سرعت در جهت قدامی خلفی و طرفی، میانگین کلی سرعت و طول مسیر بودند. با استفاده از آزمون Independent t و Paired t تأثیر خستگی در دو گروه و مقایسه وضعیت تعادل در گروه مورد بررسی قرار گرفت. یافته‌ها: در گروه کمردردی، خستگی تأثیر معنی‌داری بر هیچ کدام از پارامترها نداشت، اما در گروه سالم خستگی باعث تغییر معنی‌دار در پارامترهای مشتق از مرکز فشار شد. همچنین قبل از خستگی، ثبات وضعیتی در بین دو گروه تفاوت معنی‌داری نداشت. نتیجه‌گیری: نتایج گویای این مطلب است که سیستم کنترل وضعیتی افراد کمردردی قابل تمییز از افراد سالم نمی‌باشد مگر اینکه در معرض اغتشاشی قرار گیرد. همچنین افراد سالم استراتژی‌های متفاوتی در مقایسه با افراد مبتلا به کمردرد در مقابله با خستگی از خود نشان می‌دهند.

Title: مقایسه یادگیری حرکتی صریح و یادگیری حرکتی ضمنی در کودکان مبتلا به اوتیسم با عملکرد بالا و آسپرگر با همتایان عادی

http://jrrs.mui.ac.ir/index.php/jrrs/article/view/514

مقدمه: اختلالات طیف اوتیسم با ناهنجاری‌های مناطق قشری همراه می‌باشند که پایه‌ای برای نقص یادگیری حرکتی ایجاد کرده‌اند. یادگیری حرکتی به دو طبقه اصلی صریح و ضمنی تقسیم می‌شود. هدف این پژوهش، تعیین تفاوت یادگیری حرکتی صریح با ضمنی و میزان تحکیم یادگیری بعد از 24 ساعت در پسران مبتلا به اختلالات طیف اوتیسم و همتایان عادی بود. مواد و روش‌ها: در این مطالعه، 30 پسر مبتلا به اختلالات طیف اوتیسم با 32 پسر همتای عادی مقایسه شدند. نمونه‌های سالم و مبتلا به اختلالات طیف اوتیسم با استفاده از تست ASSQ     از دبستان‌های پسرانه شهرستان نجف آباد انتخاب گردیدند. هر دو گروه در روز اول به انجام آزمون زمان عکس‌العمل متوالی (10 بلوک) با دست راست پرداختند و روز بعد، آزمون را در 4 بلوک به منظور سنجش تحکیم انجام دادند. آزمون ANOVA با اندازه‌گیری مکرر و با استفاده از 19SPSS جهت تحلیل داده‌ها مورد استفاده قرار گرفت. یافته‌ها: دو گروه در موضوع یادگیری صریح تفاوت معنی‌داری با یکدیگر داشتند که بیانگر نقص یادگیری صریح در افراد مبتلا به اختلالات طیف اوتیسم بود (009/0 = P). نتایج همچنین عدم تفاوت معنی‌دار را در یادگیری ضمنی بین دو گروه نشان داد (385/0 = P)، به علاوه یافته‌ها حاکی از این است که تحکیم یادگیری ضمنی در گروه اختلالات طیف اوتیسم (160/0 = P) نیز بی‌نقص و تحکیم یادگیری صریح دچار اختلال (046/0 = P) می‌باشد. نتیجه‌گیری: نقص یادگیری حرکتی صریح و تحکیم آن در گروه اختلالات طیف اوتیسم می‌تواند به علت اختلال عملکرد نیمکره چپ، تخصصی شده در یادگیری صریح، باشد. به علاوه تحکیم یادگیری صریح وابسته به خواب است و در گروه اختلالات طیف اوتیسم اختلالات خواب وجود دارد، بنابراین این نقص دور از انتظار نیست. سالم بودن یادگیری ضمنی و تحکیم آن را نیز می‌توان به تأثیر نیمکره راست در یادگیری حرکتی ضمنی نسبت داد.

Title: لکنت و رسش مغزی

http://jrrs.mui.ac.ir/index.php/jrrs/article/view/477

مقدمه: تاکنون یافته‌های متعددی دال بر وجود نقایص متعدد در جنبه‌های زبانی، پردازش شنیداری و حرکتی گفتار در افراد مبتلا به لکنت گزارش شده است. تنوع این آسیب‌ها امکان گزینش فرضیه مطلوب جهت توجیه لکنت را با دشواری مواجه می‌سازد، اما به نظر می‌رسد که با مطرح شدن فرضیه رسش مغزی که به تازگی بر اساس یافته‌های الکتروآنسفالوگرافیک پیشنهاد شده است، بتوان تنوع موجود در نشانه‌های مشاهده شده را توجیه کرد. مواد و روش‌ها: این مقاله مروری نقلی بوده و تلاشی جهت توجیه یافته‌های متعدد مرتبط با لکنت در قالب فرضیه کندی رسش مغزی است. جهت نگارش این مقاله، از منابع دست اول و دست دوم با توجه به واژگان کلیدی "نوروفیزیولوژی لکنت" و "نظریه رسش مغزی" استفاده شد. جستجو در پایگاه‌های اطلاعاتی  Pubmed، Science direct، Proquest، موتور جستجوگر scholar Google و بازه زمانی سال 1990 تا 2012 و منابع کتابخانه‌ای صورت گرفت. نتایج: پس از جستجو، مقالات مربوط به لکنت نوروژنیک، سایکوژنیک و موارد مشابه از مطالعه خارج شدند. در نهایت تعداد 32 مقاله و 3 کتاب انتخاب گردید. انتخاب بر اساس ارتباط موضوعی (لکنت رشدی) و حداقل 10 برای مقالات منتشر شده از سال 1990 تا 1999 و حداقل 2 ارجاع برای مقالات منتشر شده در بازه زمانی 2010 تا 2012 صورت گرفت. 3 مورد از مقالات به صورت چکیده استفاده شد. یافته‌ها: در این مقاله ضمن تأکید بر الگوی بهبودی خودبخودی در لکنت، شواهد ناهمگرا در حمایت از فرضیه رسش مغزی مورد تحلیل قرار گرفتند. یافته‌ها حاکی از نقص در پردازش‌های زبانی، شنیداری و حرکتی در این اختلال می‌باشد. بهبودی خودبخودی به عنوان شاخصی از رسش مغزی مورد بحث قرار گرفت. نتیجه‌گیری: تنوع آسیب‌ها در لکنت می‌تواند ناشی از کندی رسش سیستم زبانی- حرکتی باشد. به نظر می‌رسد این کندی رسش مغزی سبب آشفتگی در برتری طرفی پردازش‌های زبانی- حرکتی در افراد مبتلا به لکنت می‌گردد. بررسی این فرضیه با روش‌هایی که می‌توانند رسش عصبی را چه به طور کلی و چه انتخابی افزایش دهند، مورد محک قرار بگیرد.

Title: بررسي ارتباط ناهنجاری‌های ستون فقرات با انعطاف‌پذيري و شاخص توده بدنی در دختران سندرم داون

http://jrrs.mui.ac.ir/index.php/jrrs/article/view/511

مقدمه: سندرم داون یکی از متداول‌ترین اختلالات ژنتیکی در انسان با عقب‌‌ماندگی ذهنی خفیف تا متوسط است. مهم‌ترین نشانه‌ها و عوارض این اختلال، کاهش کارکرد ذهن و ناهنجاری‌های جسمانی است که بر فعالیت‌های حرکتی آنان اثر می‌گذارد. هر چند چگونگی این اثرات منفی به درستی روشن نیست. هدف از تحقیق حاضر، بررسی ارتباط بین انعطاف‌پذیری و شاخص توده بدنی با ناهنجاری‌های ستون فقرات در افراد سندرم داون بود. مواد و روش‌ها: نمونه آماری این پژوهش شامل 50 دانش‌آموز دختر عقب‌مانده ذهنی با سندرم داون (میانگین سن 77/1 ± 96/13 سال، میانگین قد 19/8 ± 40/135 سانتی‌متر، میانگین وزن 21/9 ± 86/42 کیلوگرم، میانگین IQ 73/3 ± 12/59) است که به صورت تصادفی به عنوان آزمودنی انتخاب شدند. اطلاعات فردی، سن، قد، وزن، IQ و سوابق پزشکی با استفاده از پرونده پزشکی دانش‌آموزان سندرم داون جمع‌آوری شد. برای ارزیابی انعطاف‌پذیری از آزمون خمش و رسش استفاده شد. سپس شاخص توده بدن اندازه‌گیری گردید. در ادامه برای ارزیابی ناهنجاری‌های ستون فقرات شامل کایفوز، لوردوز و اسکولیوز پشتی و کمری از دستگاه اسپاینال موس استفاده شد. یافته‌ها: ارتباط معنی‌داری بین شاخص توده بدن با لوردوز وجود داشت (05/0 ≥ P)، اما ارتباط معنی‌داری بین انعطاف‌پذیری با هیچ یک از ناهنجاری‌های ستون فقرات گزارش نشد. نتیجه‌گیری: با توجه به ارتباط متقابل میان وضعیت بدنی و آمادگی جسمانی و نیازهای ویژه‌ای که افراد سندرم داون در اجرای بهینه فعالیت‌های حرکتی روزمره خود دارند، به مربیان توصیه می‌شود، تجویز و اجرای برنامه‌های اصلاح وضعیت، برنامه‌های بهبود آمادگی جسمانی و برنامه‌های کنترل وزن و کاهش چاقی را برای این افراد در مرکز توجه خود قرار دهند.

Title: فاکتورهای خطر همراه با کم‌شنوایی در نوزادان: بررسی نتایج 2940 نوزاد ارجاعی در برنامه غربالگری شنوایی شهر اهواز

http://jrrs.mui.ac.ir/index.php/jrrs/article/view/590

مقدمه: فراوانی کم‌شنوایی در نوزادان دارای فاکتور خطر کم‌شنوایی، 10 تا 20 برابر بیش از نوزادان بدون فاکتور خطر می‌باشد، بنابراین هدف از مطالعه حاضر، بررسی عوامل خطر کم‌شنوایی در نوزادان مراجعه کننده به طرح غربالگری شنوایی در شهر اهواز و بررسی میزان تأثیر هر یک در وقوع کم‌شنوایی بود. مواد و روش‌ها: این مطالعه از نوع توصیفی- تحلیلی- مقطعی بود. در این پژوهش پرونده‌های شنوایی مربوط به نوزادانی که در مرحله اول آزمون غربالگری شنوایی شهر اهواز رد شده بودند و دارای فاکتور خطر کم‌شنوایی بودند، از سال 1387 تا پایان 1390 مورد استفاده و بررسی قرار گرفتند. در نهایت تمام اطلاعات با استفاده از نرم‌افزار آماری SPSS و روش‌های آماری 2c و مقایسه نسبت‌ها، تجزیه و تحلیل شدند. یافته‌ها: از 2940 نوزاد مورد بررسی، 478 نفر (16 درصد) دارای یک یا تعداد بیشتری فاکتور خطر کم‌شنوایی بودند. از این تعداد در کل 52 نوزاد کم‌شنوا شناسایی شدند. بین وقوع کم‌شنوایی در گروه دارای فاکتور خطر کم‌شنوایی و گروه هنجار تفاوت آماری معنی‌داری وجود داشت (05/0 > P). سندرم‌های همراه با کم‌شنوایی و ناهنجاری‌های کرانیوفاسیال، بیشترین میزان احتمال را در وقوع کم‌شنوایی به خود اختصاص داده بود. نتیجه‌گیری: با توجه به تأثیر بالای فاکتورهای خطر کم‌شنوایی بر وقوع کم‌شنوایی، انجام آزمون‌های شنوایی در نوزادان دارای این فاکتورها به طور قطع توصیه می‌شود.

Title: ارزیابی پردازش زمانی در افراد مبتلا به صرع لوب گیجگاهی با استفاده از آزمون توالی الگوی دیرشی

http://jrrs.mui.ac.ir/index.php/jrrs/article/view/562

مقدمه: مطالعات متعددی تأثیر ضایعات مغزی بر توانایی تشخیص محرک‌های متوالی شنیداری را به ثبت رسانده‌اند. نتیجه برخی تحقیقات نشان می‌دهد که نیمکره چپ در اکثر افراد برای پردازش زمانی و به ویژه توالی زمانی غالب است. هدف از مطالعه حاضر، ارزیابی پردازش زمانی با استفاده از آزمون توالی الگوی دیرشی در افراد مبتلا به صرع لوب گیجگاهی یک طرفه بود. مواد و روش‌ها: پژوهش مقطعی- مقایسه‌ای حاضر بر روی 25 فرد مبتلا به صرع لوب گیجگاهی (11 نفر مبتلا به صرع لوب گیجگاهی نیمکره راست و 14 نفر مبتلا به صرع لوب گیجگاهی نیمکره چپ) و 18 فرد هنجار در محدوده سنی 50-15 سال با آزمون توالی الگوی دیرشی به روش پاسخگویی کلامی و زمزمه‌ای صورت گرفت. درصد پاسخ صحیح آزمون توالی الگوی دیرشی با آزمون ANOVA مورد تجزیه و تحلیل قرار گرفت. یافته‌ها: اختلاف‌های معنی‌داری بین گروه‌ها در نتایج آزمون توالی الگوی دیرشی از طریق آزمون ANOVA مشاهده شد (01/0 > P). آنالیز Tukey اختلاف‌های معنی‌داری بین گروه‌های مبتلا به صرع لوب گیجگاهی نیمکره چپ و گروه هنجار و همین طور بین گروه‌های مبتلا به صرع لوب گیجگاهی نیمکره چپ و صرع لوب گیجگاهی نیمکره راست نشان داد (05/0 > P)، اما اختلاف بین بیماران مبتلا به صرع لوب گیجگاهی نیمکره راست و گروه هنجار معنی‌دار نبود (05/0 < P). نتیجه‌گیری: بیماران مبتلا به صرع لوب گیجگاهی نیمکره چپ در پردازش توالی زمانی اختلال نشان دادند که اختصاصی بودن نیمکره چپ در پردازش ترتیب زمانی را اثبات می‌کند.

Title: بررسی روایی و پایایی خط‌کش انعطاف‌پذیر در اندازه‌گیری کایفوز

http://jrrs.mui.ac.ir/index.php/jrrs/article/view/480

مقدمه: رادیوگرافی یکی از متداول‌ترین روش‌های اندازه‌گیری کایفوز می‌باشد که از روایی و پایایی بالایی برخوردار است، اما به دلیل قرار گرفتن فرد در معرض اشعه و دربرداشتن هزینه زیاد، برای انجام معاینات دوره‌ای و غربالگری مناسب نمی‌باشد. ابزارهای کلینیکی مختلفی نیز برای این منظور وجود دارند که امکان ارزیابی سریع و ایمن انحناهای ستون فقرات را فراهم می‌کنند. هدف از انجام این مطالعه، بررسی روایی و پایایی خط‌کش انعطاف‌پذیر (Flexicurve) در اندازه‌گیری کایفوز توراسیک بود. مواد و روش‌ها: در مطالعه حاضر 105 فرد مبتلا به هایپرکایفوز در محدوده سنی 10 تا 80 سال شرکت کردند. زاویه Cobb هر یک از شرکت کنندگان توسط پزشک متخصص ستون فقرات اندازه گرفته شد. دو معاینه کننده دیگر که اطلاعی از نتایج زاویه Cobb نداشتند، توسط خط‌کش انعطاف‌پذیر، زاویه کایفوز توراسیک بیماران را اندازه می‌گرفتند. یافته‌ها: مقایسه زاویه کایفوز اندازه‌گیری شده توسط خط‌کش انعطاف‌پذیر با زاویه Cobb نشان می‌دهد که روایی این ابزار ضعیف می‌‌باشد. همچنین نتایج این آزمون نشان داد که خط‌کش انعطاف‌پذیر دارای پایایی درون فردی قابل قبولی در اندازه‌گیری کایفوز در هر دو محدوده سنی زیر30 سال و بالای 50 سال می‌باشد و با وجود آن که پایایی بین فردی این ابزار در محدوده سنی زیر 30 سال ضعیف است، اما در محدوده سنی بالای 50 سال از پایایی بین فردی قابل قبولی برخوردار می‌باشد. نتیجه‌گیری: خط‌کش انعطاف‌پذیر از روایی ضعيفي در اندازه‌گيري كايفوز توراسيك برخوردار مي‌ باشد. بنابراین با وجود آن که این روش بسیار ساده، ایمن، غیر تهاجمی و مقرون به صرفه است، اما به دلیل آن که از پایایی و روایی بالایی برخوردار نیست، استفاده از آن در معاینات دوره‌ای و مطالعات غربالگری توصیه نمی‌گردد.

Title: ارتز رباتيک مچ- کف پايي با حسگر لامسه جهت کنترل هوشمند راه رفتن افراد مبتلا به افتادگي مچ پا

http://jrrs.mui.ac.ir/index.php/jrrs/article/view/479

چكيدهعارضه افتادگي پا را می‌توان بيماري عصبي، عضلاني و ناشي از نقص ژنتيکي، جراحات جنگي، تصادف، رشد غده‌ها و برخي سکته‌هاي مغزي ذکر کرد که در اثر آن عصب پا دچار آسيب ديدگي دایم يا برگشت‌پذير مي‌شود. در اثر اين عارضه شخص مجبور مي‌شود براي جبران اين نقص که موجب ناتواني در باز و بسته ‌کردن مچ پا مي‌شود، از ارتز استفاده کند. يا اين که مشکل لنگيدن پا براي وي به وجود خواهد آمد. دستگاه ارتز رباتيک هوشمند شامل صفحه حسگر فشار کف پا، نرم‌افزار محاسبه‌گر و بخش ربات متحرک است. با توجه به مدل ساخته شده بر اساس اطلاعات حرکتي افراد دچار افتادگي پا مشخص شد که نياز به سامانه رباتيکي است که بتواند ميزان گشتاور مورد نياز بيمار را براي راه رفتن بهبود بخشد و به همين دليل طراحي ارتز جديدي لازم بود که بر اساس سنجش فشار کف پا در شرايط گوناگون راه رفتن بتواند ميزان گشتاور جبراني مورد نياز را براي سامانه رباتيک تشخيص دهد و توسط نيروي الکتريکي تأمين نمايد. براي ايجاد ارتباط بين اين سامانه تأمين گشتاور و فشار سنجش شده از فشارسنج از مدل‌هاي هوشمند استفاده شده است. اين سامانه باعث بهبود عملکرد و رفع ناتواني فرد دچار افتادگي پا مي‌شود و توانايي شخص را در راه رفتن بهبود مي‌بخشد.کاهش فشار و آسيب به تاندون‌ها، کاهش ضربه به مفاصل، کمک به بهبود بيماري با استفاده از سامانه رباتيک و جبران ضعف عملکرد عضلات، از جمله اهدافي پيش‌بيني شده براي ساخت دستگاه بود.
